# Delivery of Community-Based Palliative Care: Findings from a Time and Motion Study

**DOI:** 10.1089/jpm.2016.0433

**Published:** 2017-10-01

**Authors:** Nrupen A. Bhavsar, Kate Bloom, Jonathan Nicolla, Callie Gable, Abby Goodman, Andrew Olson, Matthew Harker, Janet Bull, Donald H. Taylor

**Affiliations:** ^1^ Division of General Internal Medicine, Department of Medicine, Duke University School of Medicine, Durham, North Carolina.; ^2^ Duke Clinical Research Institute, Duke University, Durham, North Carolina.; ^3^ Duke Cancer Institute, Duke University, Durham, North Carolina.; ^4^ Duke University, Durham, North Carolina.; ^5^ Margolis Center for Health Policy, Duke University, Durham, North Carolina.; ^6^ Four Seasons, Flat Rock, North Carolina.; ^7^ Sanford School for Public Policy, Duke University, Durham, North Carolina.

**Keywords:** hospice, palliative care, time and motion

## Abstract

***Background:*** Use of palliative care has increased substantially as the population ages and as evidence for its benefits grows. However, there is limited information regarding which care activities are necessary for delivering high-quality, interdisciplinary, community-based palliative care.

***Objectives:*** This study aims to identify and measure the discrete clinical and administrative activities completed by a multidisciplinary team in a hospice provider-led model for providing community-based palliative care.

***Study Design:*** A time and motion study was conducted at three care settings within a large hospice and palliative care network and a process map was drawn to describe the personnel and activities recorded.

***Methods:*** Researchers recorded activities performed by clinical and administrative staff. Activities were categorized into those related to patient care, administrative duties, care coordination, and other. A process map of palliative care delivery was created and descriptive statistics were used to calculate the proportion of time spent on discrete activities and within each activity category.

***Results:*** Over 50 hours of activities were recorded during which the clinicians interacted with 25 patients and engaged in 20 distinct tasks. Physicians spent 94% of their time on tasks related to patient care and 1% on administrative tasks. Nurse practitioners and registered nurses spent 82% and 53% of their time on patient-related tasks and 2% and 37% on administrative tasks, respectively.

***Conclusion:*** The delivery of palliative care is interdisciplinary and involves numerous discrete tasks and activities. Understanding the components of a community-based palliative care model is the first step to designing incentives to encourage its spread.

## Background

Palliative care is an interdisciplinary, coordinated delivery approach that focuses on improving the quality of life of individuals with life-limiting illness through relief from symptoms, pain, and the stress of serious illness.^1^ Research has shown that palliative care can improve quality of life and, at times, survival in patients with life-limiting illness.^2,3^ Use of palliative care has increased substantially over the past few years as research supports its benefits and as the US population ages.^4^ However, as an emerging discipline, there is wide variation in how providers deliver palliative care. Limited information is available on the discrete tasks and activities involved in the delivery of high-quality, community-based palliative care, which would identify what resources would be needed to expand community-based palliative care more broadly.

The main models of palliative care delivery include hospitals, long-term care facilities, or organizations such as hospice providers that offer community-based programs. The most common setting for nonhospice palliative care in the United States has been hospitals.^4^ More recently, the use of community-based palliative care has increased, driven in large part by policy-makers' and health system stakeholders' search for alternative payment approaches that increase the value for healthcare purchasing—a trend accelerated by the passage and implementation of the Affordable Care Act.^5^ Community-based palliative has been shown to improve quality of care and patient outcomes while potentially reducing costs.^6^ However, current fee-for-service payment approaches used by Medicare and other payers do not incentivize the provision of this care.

This article fills a void in the literature by using time and motion methods to describe a community-based palliative care model operated by a nonprofit hospice provider, Four Seasons Compassion for Life, which serves patients in western North Carolina. The model is designed to deliver palliative care to patients upstream of hospice election and help patients and their families address symptoms of life-limiting illness and make healthcare decisions. Patients enter the model through a physician or facility referral and are then cared for by a team comprising clinical and administrative staff and volunteers. The palliative care team delivers care to patients and their families across home, clinic, and facility care settings, depending on their needs, and follows patients as they transition across these settings. The purpose of this article is to describe the model as it was implemented over the course of two days of observation.

Characterizing the clinical and administrative activities that constitute the model is an important step toward understanding the resources needed to provide such care in other places. The Four Seasons palliative care model is being evaluated as part of a Center for Medicare & Medicaid Innovation (CMMI) HCIA-Round Two Innovation Award to determine if the care provided through this approach can reduce cost to the Medicare program while at least holding quality of care constant. A team of Duke researchers, including some of the authors of this study, will use the results of the demonstration to design an alternative payment model for upstream palliative care. Given the high-profile nature of the CMMI Innovation Award program, this model of palliative care is an important one to fully describe since it will be a natural comparator for other palliative care models that will necessarily be idiosyncratic with respect to care team and structure of care provision. The purpose of this article is to describe the model as care was delivered over the course of two days.

A prospective time and motion study was conducted to identify and measure each of the tasks and activities carried out by the Four Seasons team while delivering care in their model. Time and motion studies are a quantitative data collection method whereby external observers collect detailed information on activities performed and the amount of time spent to conduct those activities.^7^ They originated in other disciplines, but time and motion studies have recently been used in healthcare to study healthcare delivery and ways to improve patient safety and quality.^7–9^ As part of this time and motion study, a process map was created to visualize a series of discrete tasks and activities completed in the delivery of palliative care. Process maps have also been used in engineering and business to identify the series of steps that must be completed to achieve a result.^10^ More recently, it has been used in healthcare to understand patient care.^11–13^ The results will serve as the foundation for understanding what resources are needed to deliver the model and how they could be financed through an alternative payment model to support a palliative care service line that traditionally runs at a loss under the current reimbursement structure that values procedures over time spent with patients and families.

## Methods

### Study design

A time and motion study was conducted at three locations within the Four Seasons Compassion for Life hospice and palliative care network. Four Seasons is located in Flat Rock, North Carolina, and is one of the largest providers of hospice and palliative care services in western North Carolina. Annually, they provide care to 5000 palliative care and hospice patients. The study was conducted within specific locations within the network, including the Four Seasons Compassion for Life facility, an outpatient palliative care clinic, and a distinct home setting. A team of five researchers from the Duke University Medical Center were invited to observe and record activities and tasks performed by one physician, two nurse practitioners, two registered nurses, two social workers, three administrative staff, and one volunteer over the course of two days. The days chosen were based on mutually convenient days for the researchers and palliative care team. The providers and staff included in the study were team members who were scheduled to work those days. Data collected by each researcher were collated to create an accurate accounting of time spent by clinicians on each patient during the observation days. The study was approved by the Institutional Review Board (IRB) at Duke University.

### Data collection

A data collection form was created to track clinician, administration, and social worker activities and tasks. The form included predefined providers and tasks, but included space for free-form text. The form was pilot tested by researchers within an outpatient palliative care setting. Iterative changes were made to improve usability and feasibility of the form. On site, researchers recorded observations and used semistructured interview techniques to obtain an accurate depiction of the discrete processes involved in the delivery of palliative care. Researchers calculated the time spent on each activity/task by recording the start and end times of each activity and task using digital clocks. Due to the nature of the Institutional Review Board (IRB) approval, researchers did not directly witness patient/physician interactions.

Individuals involved in the care of patients were categorized as clinicians and administrative staff. Clinicians included physicians, nurse practitioners, registered nurses, and social workers. Administrative staff included the palliative care team administrative (PCTA) staff and the palliative care program specialist (PCPS) and a volunteer. The specific activities/tasks performed were categorized into patient visits, administrative tasks, care coordination, and other.

### Process map

A process map of the Four Seasons model of community-based palliative care delivery was created by summarizing the discrete activities performed by the care team's clinical and administrative staff. Items on the process map were divided into tasks (depicted as squares) and decisions (depicted as diamonds). Activities and tasks were organized by the three locations of palliative care delivery: facility, clinic, and home.

### Statistical analysis

Descriptive statistics were used to calculate the proportion of time spent on individual activities and tasks, as well as task categories.

## Results

During the course of the study, we recorded 50.5 hours of activities. In total, the care team interacted with 25 patients either in person or over the phone. A total of 20 distinct tasks were identified.

Each of the team members and the task and activities they performed are listed in Table 1. Tasks are organized by patient care, administrative, and other and are cross-walked with the palliative care team staff member(s) who completed each task. The palliative care team included a medical doctor (MD), nurse practitioners (NPs), registered nurses (RNs), social workers (SWs), palliative care program specialists (PCPSs), patient care team administrators (PCTAs), and a volunteer (V).

**Table 1.** List of Task Categories, Team Members, and Specific Task in the Delivery of Community-Based Palliative Care

*Task category*	*Team member*	*Task/activity*
Patient care	MD, SW, PCPS, *NP, RN, V*	Initial patient visit
	MD, NP, RN, SW, PCPS, V	Subsequent patient visit
	MD, NP, RN, SW, PCTA, PCPS	Charting
	NP, *MD, PA, RN, SW, PCTA*	QDACT
	MD, NP, RN, SW, PCTA, PCPS	Phone call
	RN	Initial patient phone assessment
	MD, NP, SW	Preparing for patient visit
	MD, NP, RN, SW, PCPS	Prescription management/discussion
	NP, RN, PCTA	MD/RN support
	MD, NP, RN, SW, PCPS	Discussions with other staff about patients (in person)
	PCTA, *MD, NP*	Hospital checkout
	RN, PCTA, *MD, NP*	Hospice transition
	NP, RN	Prepare patient for MD/NP
	PCTA	IDG Reports
Administrative	MD, NP, PCTA, *RN, SW*	Billing
	MD, PCTA, PCPS, V	Scheduling
	PCPS, PCTA, *MD, NP, RN, V*	General administrative tasks
	PCTA, PCPS	Caseload generation
	RN, PCTA, *MD, NP, SW, V*	Meetings
Other	MD, NP, RN, PCTA, PCPS, V	Other

Italicized team members engage in tasks/activities listed based on interviews, but were not observed.

IDG, interdisciplinary Group; MD, physician; NP, nurse practitioner; PCPS, palliative care program specialist; PCT Admin, palliative care team administrative staff; PQRS, physician quality reporting system; QDACT, quality data assessment tool; RN, registered nurse; SW, social worker; V, volunteer.

During the two days of the study, physicians were observed for 617 minutes, nurse practitioners for 704 minutes, registered nurses for 456 minutes, and social workers for 460 minutes (Table 2). Over this time period, physicians spent 94% of their time on patient care tasks, 1% on administrative tasks, and 4% on other tasks. Nurse practitioners spent 82% of their time on patient care tasks, 2% on administrative tasks, and 16% on other tasks. The registered nurses spent 53% of their time on patient care tasks, 37% on administrative tasks, and 10% on other tasks. Social workers spent 100% of their time on patient care tasks. The administrative staff spent the greatest proportion of their time on administrative tasks with 70% for PCTAs and 38% PCPSs.

**Table 2.** Proportion of Observed Time Spent on Each Task Category Over two Days

	*Task categories*			
*Provider*	*Total time (minutes)*	*Patient care (%)*	*Administrative tasks (%)*	*Other (%)*
Clinical staff				
MD	617	94	1.3	4.3
NP	704	82	2.0	16
RN	456	53	37	10
SW	460	100	—	—
Administrative staff				
PCTA	564	27	70	2.8
PCPS	278	37	38	25
Volunteer	22	9	36	55

Proportion of time spent on tasks may not add up to 100% due to rounding.

Table 3 lists the proportion of time the clinical and administrative staff spent on individual tasks. Physicians spent the majority of their time on tasks related to the initial patient visit and charting (24.8% and 24.1%, respectively). Nurse practitioners spent the majority of their time on tasks related to the subsequent patient visit (29.0%) and phone calls (22.3%). Registered nurses spent the majority of time in meetings (37.3%) and in support to other clinicians (15.4%). Social workers spent the majority of their time on tasks related to the initial and subsequent patient visits (32.8% and 38.7%, respectively). The administrative staff spent the majority of their time in meetings (PCTAs: 51.5%). Outside of uncategorized tasks, the PCPSs and volunteer spent the majority of their time on scheduling (20.1% and 36.4%, respectively).

**Table 3.** Percentage of Time Spent by Staff on Individual Tasks

		*Clinical staff*				*Administrative staff*		
	*Task*	*MD*	*NP*	*RN*	*SW*	*PCTA*	*PCPS*	*Volunteer*
Patient care	Initial patient visit	24.8	0	0	32.8	0	12.2	0
	Subsequent patient visit	19.4	29	6.1	38.7	0	9.0	9.1
	Charting	24.1	12.4	6.1	12	10.3	0.4	0
	QDACT	0	1.3	0	0	0	0	0
	Phone call	6.5	22.3	8.3	3.9	1.6	14.4	0
	Patient phone assessment	0	0	2.9	0	0	0	0
	Preparing for patient visit	0.8	4.4	0	5.4	0	0	0
	Prescriptions	14.4	2.4	10.1	1.7	0	0.4	0
	MD/RN support	0	5.1	15.4	0	5.1	0	0
	Discussions with other staff about patients (in person)	4.2	4.5	3.7	5.4	0	0.4	0
	Hospital checkout	0	0	0	0	2.5	0	0
	Hospice transition	0	0	0.4	0	5.1	0	0
	Preparing patient for MD/NP	0	0.7	0.7	0	0	0	0
	IDG Reports	0	0	0	0	2.8	0	0
Administrative	Billing	0.6	2	0	0	6.2	0	0
	Scheduling	0.6	0	0	0	2.5	20.1	36.4
	General administrative tasks	0	0	0	0	7.0	4.3	0
	Caseload generation	0	0	0	0	2.7	13.7	0
	Meetings	0	0	37.3	0	51.5	0	0
other	Other	4.3	15.9	9.6	0	2.8	25.2	54.5

Proportion of time spent on tasks may not add up to 100% due to rounding.

### Components of palliative care delivery

**FIG. 1.**
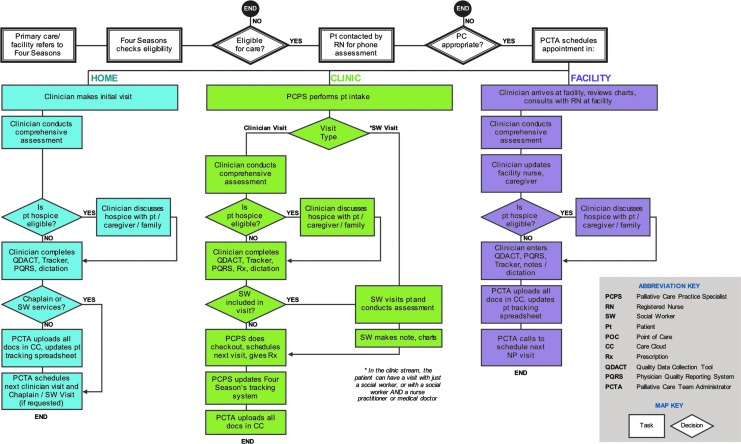
Process map of community-based palliative care delivery.

A process map was created to visualize how palliative care is delivered by Four Seasons (Fig. 1). A patient enters the Four Seasons care model through a referral source such as their primary care provider. The Four Seasons staff then assesses the patient's eligibility for palliative care based on health status, diagnosis, and active comorbidities. A registered nurse (RN) then conducts a phone assessment on eligible patients and stratifies patients based on their risk profile. If palliative care is deemed appropriate, the PCTA schedules the patient for an appointment with a Four Seasons clinical provider, who is able to see a patient at the Four Seasons' facility, a nursing home facility, at an outpatient palliative care clinic, or at the patient's home.

The subsequent elements of care and pathway are unique to care setting, but with much overlap across all settings. Initially the clinician, who can be a physician, a nurse practitioner, or a registered nurse, conducts a comprehensive assessment, where the clinician collects information on the patient's current conditions and immediate medical needs, including advanced directives, symptom review, goals of care discussion, assessment of psychosocial and spiritual needs, and overall well-being. At the initial patient assessment, the clinician completes the Quality Data Assessment Tool (QDACT), which includes symptom scores, advance care planning, and prognostication. Included in QDACT are elements to fulfill the physician quality reporting system (PQRS) requirement. If the patient has a prognosis of less than six months and is appropriate and desirous of hospice, a referral is sent to the administrative staff who uploads this information to the Four Seasons server. A hospice RN is sent out to complete the admissions process. If the patient is not appropriate for hospice, the clinician determines the risk stratification level, which determines the visit frequency from palliative care team members. Risk stratifications have been developed for clinical, psychosocial, and spiritual assessments.

## Discussion

This study quantifies the discrete tasks involved in the delivery of community-based palliative care and the personnel involved in the delivery of this care. It is important to contribute to a descriptive process of what palliative care is outside of specialized inpatient settings. By leveraging time and motion methods and process mapping, we visualized the process of delivering community-based palliative care and found that the delivery of community-based palliative care at a single institution over a discrete time frame requires 20 broadly defined tasks performed by 11 different clinical and administrative staff. We found that clinicians may spend the majority of their time on tasks related to patient care, which may not include direct interactions with patients. Certain clinicians spend a significant amount of time on administrative tasks and tasks related to the coordination of care. Leveraging administrative support staff to complete some of these tasks may provide clinicians more time to interact with patients, which is the highest value of service in palliative care delivery.

To our knowledge, this is the first time and motion study to measure the processes of a community-based palliative care model in the United States. Time and motion studies have been performed in a variety of business settings to better understand business models and improve efficiency. More recently, time and motion analysis has been applied to the healthcare industry to improve care delivery and efficiency in the areas of oncology, surgery, pediatrics, nephrology, and intensive care.^14–19^ Past work has focused on mapping the workflow of facilities and determining the impact of individual tasks that may improve patient outcomes and quality of care, and potentially reduce costs.^20–22^ While time and motion methods have not been used extensively in palliative care research, a limited number of studies have leveraged these methods to examine resource use and costs associated with managing symptoms and different therapies.^23,24^ Our study extends the field by applying time and motion methods to identify and measure the discrete tasks and activities involved in the delivery of a community-based palliative care model.

Similarly, process mapping is a methodology that has useful health applications, but has traditionally been utilized primarily in engineering and manufacturing, two disciplines heavily reliant on procedure.^13,25^ A review of process mapping in healthcare shows that the technique has been used in three settings: operating rooms, specimen handling, and reducing patient wait times.^25–31^ Within these settings, process mapping has been primarily utilized for process efficiency, process discovery, or resource efficiency. While there is significant evidence for the use of process mapping across many disciplines throughout healthcare, there is little evidence that process mapping has been used in palliative care. A 2015 literature review of process mapping within palliative care reviewed 2379 publications that mentioned strategies to improve the organization of palliative care and found only one publication that highlighted process mapping.^32^ Due to the lack of standardization within palliative care, little research has been conducted that quantifies the usage of process mapping within palliative care. As an emerging discipline, best practices in the delivery of effective palliative care are still widely nonuniform. There appears to be variability in palliative care delivery from institution to institution and even provider to provider. Our study attempts to add structure to the delivery of community-based palliative care.

### Strengths and limitations

An important strength of this study is the use of nontraditional methods in end-of-life research (i.e., time and motion study and process mapping) to reliably record the tasks and personnel involved in the delivery of community-based palliative care. As a part of an ongoing CMMI project that aims to create new payment models for palliative care, this is an important initial step. The methods applied here should be generalizable to other palliative care settings and organizations.

An important limitation of this study is that observations made were at a single institution over the course of two days and may not represent the experience of the Four Seasons team on average. In addition, due to the nature of the IRB approval, the research team was also not able to directly witness patient/physician interactions, including the home-based provider process, which accounts for a large number of patients at Four Seasons. Instead, semi-structured interviews of the providers were conducted to enumerate the tasks they performed during home visits. However, this work is an important first step in quantifying the discrete activities involved in the delivery of palliative care.

The population at Four Seasons comprises more than 50% noncancer community-based palliative care patients. We recognize that this is not the norm at other institutions and settings. The tasks and personnel involved in palliative care delivery in cancer compared with noncancer palliative care patients may differ. We were limited by our IRB from ascertaining the patients seen during our study. In the future, we hope to gather relevant summary information about the patients themselves to compare care delivery for patients with different conditions.

## Conclusion

There is increasing demand for palliative services in the growing population of patients with life-limiting illness. This study is the first to our knowledge that quantifies the tasks and personnel involved in community-based palliative care delivery. The novel application of these methodologies to a community-based palliative care model has yielded insights that can inform future research, provider decision making, and policy-making. Future studies could use the same methods to evaluate and report on other models not only of community-based palliative care but those delivered by hospitals and long-term care facilities as well. Describing and comparing a variety of palliative care models through rigorous research could promote the development and sharing of best practices that would improve care delivery to patients and provide foundational data and information for the construction of alternative models of paying for palliative care. Despite its benefits and cost-saving potential to Medicare and other payers, palliative care is insufficiently reimbursed under the predominant fee-for-service approach that values procedures over time spent with patients. For policy-makers interested in establishing a value-based payment model to reimburse for palliative care, such as a per diem rate or bundled payment, it is essential to identify which resources and activities would need to be covered by the payment, and the results of this study help answer that question.

Funding for this article was made possible, in part, by the Centers for Medicare & Medicaid Innovation (CMMI) through grant 1C1CMS331331.Views expressed in written materials or publications and by speakers and moderators do not necessarily reflect the official policies of the Department of Health and Human Services, nor does any mention of trade names, commercial practices, or organizations imply endorsement by the US Government.
